# Holstein phonons and magnetic fields synergistically tune thermoelectric performance in dice lattices

**DOI:** 10.1038/s41598-026-52225-z

**Published:** 2026-05-09

**Authors:** Tayebeh Kakavandi, Hamed Rezania, Farshad Azizi

**Affiliations:** 1 Department of Physics, Razi University, Kermanshah, Iran; 2 Department of Physics, Jundi-Shapur University of Technology, Dezful, Iran

**Keywords:** Dice lattice, Thermoelectric performance, Holstein phonons, Electron-phonon coupling, Magnetic field, Green’s functions, Materials science, Physics

## Abstract

In this study, we investigate the transport and thermoelectric properties of a doped dice lattice subjected to electron-phonon coupling via Einstein phonons and an external perpendicular magnetic field. The electronic band structure is modeled using the Holstein Hamiltonian augmented with a Zeeman interaction term. Employing a one-loop approximation for the electronic self-energy within a full-band framework, we derive the interacting Green’s functions to examine the temperature-dependent electrical and thermal conductivities, as well as the energy-dependent density of states (DOS). Particular attention is given to the behaviors of the Seebeck coefficient, power factor, figure of merit, and Lorenz number. Our findings reveal that increasing the magnetic field diminishes the DOS at the Fermi energy, enhancing the semiconductor-like characteristics of the system. Furthermore, activating electron-phonon coupling results in a notable reduction of the DOS at zero energy. These results underscore the pivotal influence of electron-phonon interactions and magnetic fields in modulating the electronic and thermoelectric attributes of two-dimensional dice lattices, providing valuable insights for the design of advanced thermoelectric materials and devices.

## Introduction

Extensive theoretical and experimental investigations have been conducted on substances featuring honeycomb lattice arrangements, including graphite in three dimensions, graphene in two dimensions, and carbon nanotubes in one dimension^1–5^. Analogous to the charge carriers in graphene, certain low-dimensional systems exhibit quasiparticle spectra resembling relativistic behavior, governed by the Dirac equation. Except for an additional zero-energy flat band, the energy-momentum dispersion for low-energy excitations in these systems exhibits linearity^6,7^. Topological insulators^8^ and Weyl semimetals^9,10^ are two examples of such systems. One well-known example of a material with linear dispersion in its low-energy excitation spectra is the dice lattice^11^. Three separate bands make up the dice lattice model’s electrical band structure, which has three atomic sublattices per unit cell^12,13^.

As demonstrated by the SrTiO$_{3}$/SrIrO$_{3}$/SrTiO$_{3}$ heterostructure^14^, the dice lattice may be created via the epitaxial growth of cubic lattice trilayers along the [111] orientation. This lattice incorporates an additional atom at the center of each hexagon, resulting in a nondispersive flat band that crosses the two Dirac bands at the Dirac point. Coulomb interactions can easily dominate the bandwidth due to the flat band’s lack of dispersion, which may lead to the emergence of unusual quantum states like the fractional quantum Hall effect^15,16^ and high-temperature superconductivity^17^. Because of its vanishing group velocity, the flat band has no effect on electronic transport properties in the single-particle approximation. In systems that have chiral symmetry, flat bands and Dirac bands often coexist because of an imbalanced atom in the primitive unit cell. Tasaki’s ornamented square lattice^18^, the Lieb lattice^19^, and the dice lattice^20^ are notable examples. In recent years, numerous investigations have examined a variety of physical phenomena in the dice lattice, encompassing Klein tunneling^21,22^, magnetoplasmons^23^, wave packet dynamics^24^, topological phase transitions induced by Floquet driving^25,26^, and Ruderman-Kittel-Kasuya-Yosida (RKKY) interactions in related spin-polarized nanostructures^27^.

The contributions of electrons and phonons significantly influence the electrical and thermal transport characteristics in solid materials^28^. The electron-phonon coupling is one of the most important interactions that shapes the characteristics of materials. In this interaction, lattice vibrations quantized as phonons interact with electrons. One example of such interactions is Kohn anomalies, where the screening of lattice oscillations is abruptly altered due to electron-phonon mediation at the Fermi surface^29^. According to earlier analyses, electron-phonon effects are also crucial for permitting high-temperature superconductivity in graphene-derived frameworks^30^. The Holstein formalism^31,32^ captures how lattice distortions symmetrically relate to charge carrier density through ripple forms in graphene-like materials, enabling electron interactions with dispersionless out-of-plane phonons. More broadly, lattice dynamics exert a substantial influence on graphene-based materials, exemplified by the pronounced impact of localized electron-phonon coupling in magnetically ordered states on the thermal conductivity of single-layer graphene^33^. Our previous research has systematically explored the ramifications of electron-phonon coupling on various physical attributes of systems derived from graphene^34–41^, including RKKY interactions in doped carbon nanotubes.

Anisotropy affects thermal transport characteristics in Heisenberg antiferromagnets^42^, and similar effects have been shown in magnetic models on honeycomb lattices. This offers a comparative context for comprehending phonon-mediated phenomena in two-dimensional systems. The physical properties of materials that resemble graphene structures are significantly influenced by electron-phonon coupling, which is the interaction between electrons and lattice vibrations. Phenomena such as high-temperature superconductivity in bilayer graphene^30^ need this connection. The Holstein Hamiltonian^43–46^ effectively describes the symmetric distortions induced by ripple formations in the lattice, which connect to charge carriers and involve electron engagements with nondispersive out-of-plane phonons. These localized couplings at the boundaries of the Brillouin zone generate Kohn anomalies in the phonon spectrum, thereby affecting transport behaviors that can be detected through Raman spectroscopy^47,48^.

In condensed matter physics, examining the alterations in the transmission spectrum relative to the incident spectrum–termed optical absorption–has long served as a vital method for probing the structural and physical features of solid materials. Investigations have addressed the optical conductivity of single-layer graphene within the visible spectrum^37^ and across photon energies ranging from 0.2 to 1.2 eV^38^. Various studies have explored how Coulomb interactions among electrons and phonon contributions impact the optical absorption in monolayer graphene^49–51^. The interaction between intraband and interband transitions in the optical absorption of graphene has been examined by Kin Fai Mak and associates^52^. Furthermore, the optical properties of several graphene-analogous systems have been assessed and interpreted using density functional theory^53^. Additionally, beyond graphene and its derivatives, a large number of theoretical and experimental studies have investigated optical absorption in honeycomb-structured materials^54–59^.

Advancements in fabrication techniques have enabled the incorporation of expansive graphene films into optoelectronic systems, such as transparent electrodes and photodetectors^60–62^. Plasmonic devices using graphene have been investigated for sensor technologies because of its tunable optical properties^63,64^. High-speed photodetectors and wideband optical modulators^65,66^ further demonstrate graphene’s versatility in fast photonic applications. Innovative transistor topologies have been made possible by the improvement of light-matter interaction achieved by combining graphene with microcavities^67^. Computational modeling of field-effect transistors based on graphene nanoribbons has indicated potential for energy-efficient electronics^68^. Moreover, heterostructures formed via van der Waals assembly, incorporating graphene alongside other two-dimensional layers, have expanded opportunities for sophisticated optoelectronic systems^69,70^.

The dice lattice uniquely combines two linear Dirac bands with a dispersionless flat band, leading to van Hove singularities and a high DOS peak at the Dirac point that is highly tunable by Zeeman splitting and polaronic renormalization–features absent in Lieb or square lattices. This enables non-monotonic tuning of $S$ and $ZT$ near half-filling, constituting the central innovative insight of the present work.

In this study, we examine the effects of electron-Holstein phonon coupling strength, external perpendicular magnetic field, and electronic doping concentration on selected transport properties of the dice lattice at finite temperatures. The doped regime is defined by the system’s electron concentration, wherein a positive chemical potential signifies an electron density exceeding that of half-filling. Specifically, we investigate the temperature dependence of thermal and electrical conductivities in the dice lattice under different magnetic field intensities and electron-phonon coupling strengths. Additionally, we examine how the density of states behaves in response to the applied magnetic field and electron-phonon interaction. In order to get these electronic properties, we calculate the electronic self-energy using a one-loop approximation in a full-band approach. We derive the interacting electronic Green’s functions for the doped dice lattice by utilizing this self-energy. Charge structure factors in doped armchair nanotubes under electron-phonon interactions have been calculated using similar methods^71^, demonstrating the adaptability of Green’s functions in capturing correlation effects in low-dimensional carbon-based systems. The Green’s function formalism is employed to evaluate both the density of states and the transport properties. Additionally, we discuss and interpret how electron-phonon coupling influences the temperature-dependent profile of the Seebeck coefficient, a fundamental thermoelectric parameter in the dice lattice.

## Model and method

**Fig. 1:**
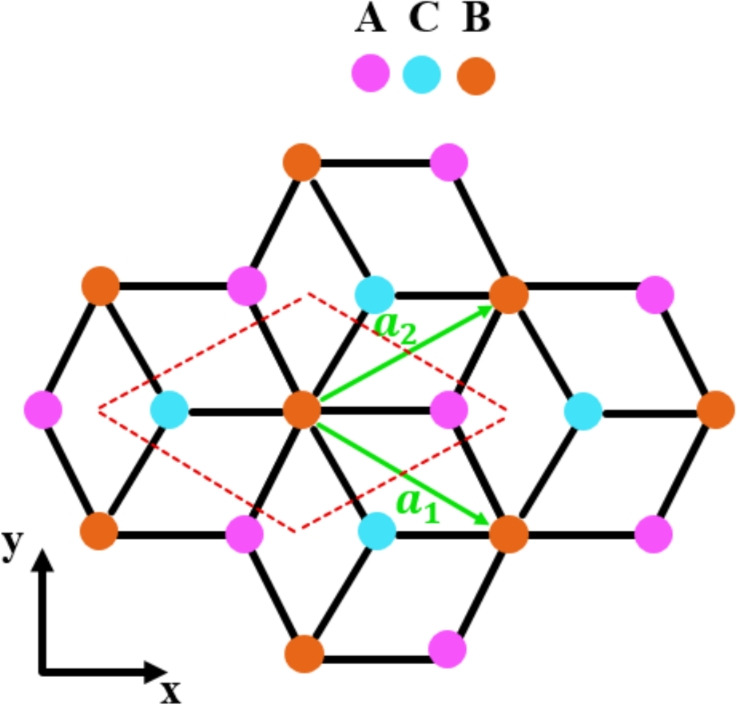
The lattice configuration of the dice structure, featuring three basis atoms per unit cell: $A$, $B$, and $C$. The primitive vectors defining the unit cell are denoted as $\textbf{a}_{1}$ and $\textbf{a}_{2}$.

The structure of the dice lattice, depicted in Fig. 1, consists of three distinct sublattices with varying on-site potentials. Each unit cell encompasses three fundamental atomic sites.

This lattice is formed by the following two basis vectors:

1 $$\begin{aligned} \textbf{a}_1 = a \left( \frac{\sqrt{3}}{2} \hat{x} + \frac{1}{2} \hat{y} \right) , \quad \textbf{a}_2 = a \left( \frac{\sqrt{3}}{2} \hat{x} - \frac{1}{2} \hat{y} \right) , \end{aligned}$$

where $\hat{x}$ and $\hat{y}$ are the unit vectors in the $x$- and $y$-axes, respectively, and $a$ is the lattice spacing. We use a tight-binding approximation augmented by electron-phonon couplings modeled using the Holstein approach^72^, which takes into account the connection between site-specific phonon modes and electron states, to investigate the electronic behavior of single-orbital electrons inside the lattice atoms. The Holstein phonons, on the other hand, which originate from perpendicular lattice oscillations, offer an appropriate way to simulate electron-lattice site interactions inside the dice model. Distinct on-site potentials are assigned to accommodate the sublattice variations.

We adopt the nearest-neighbor hopping $t = 1$ eV and sublattice potential difference $\Delta = 0.1t$, values that are directly benchmarked against DFT calculations on SrTiO$_3$/SrIrO$_3$ heterostructures^14,73^.

The Holstein Hamiltonian, spin-resolved and including a vertical magnetic field $\textbf{B} = B \hat{z}$, takes the form:

2 $$\begin{aligned} H= & -t \sum _{\langle i,j \rangle ,\sigma } \left( d^{\sigma \dagger }_{j} e^{\sigma }_{i} + h.c. \right) -t \sum _{\langle i,l \rangle ,\sigma } \left( f^{\sigma \dagger }_{l} e^{\sigma }_{i} + h.c.\right) +\sum _{i,\sigma }(2 \mu _B B \sigma - \mu ) \left( d^{\sigma \dagger }_{i} d^{\sigma }_{i}+ e^{\sigma \dagger }_{i} e^{\sigma }_{i} + f^{\sigma \dagger }_{i} f^{\sigma }_{i} \right) \nonumber \\+ & g \sum _{i,\sigma } \left( d^{\sigma \dagger }_{i} d^{\sigma }_{i}+ e^{\sigma \dagger }_{i} e^{\sigma }_{i} + f^{\sigma \dagger }_{i} f^{\sigma }_{i} \right) \left( q_{i} + q^{\dagger }_{i} \right) + \sum _{i} \omega _0 q^{\dagger }_{i} q_{i}. \end{aligned}$$

Here, $t$ denotes the nearest-neighbor hopping parameter in the honeycomb network. Within this model, the operator $e^{\sigma }_{i}$ annihilates an electron of spin $\sigma = \uparrow , \downarrow$ at the $i$-th unit cell on sublattice B. In contrast, $d^{\sigma \dagger }_{j}$ ($f^{\sigma \dagger }_{l}$) creates an electron on sublattice A (C) in the neighboring unit cell $j$ ($l$). For the initial term in Eq. (2), the position differences between adjacent unit cells are $\textbf{R}_{j} - \textbf{R}_{i} = 0, -\textbf{a}_1, -\textbf{a}_2$. Similarly, for the second term, these differences are $\textbf{R}_{l} - \textbf{R}_{i} = 0, \textbf{a}_1, \textbf{a}_2$. The on-site potentials for sublattices A, B, and C are $\Delta$, 0, and $-\Delta$, respectively. Upon reaching half-filling, the chemical potential $\mu$ is zero. Furthermore, the Bohr magneton $\mu _B$ influences spin-split energy changes by accounting for the Zeeman splitting brought on by the magnetic field.

The present Hamiltonian includes only the Zeeman term; orbital effects via Peierls substitution are neglected as they require significantly larger system sizes for convergence. Orbital quantization would further split the flat band and may enhance thermoelectric efficiency at high fields.

With magnetic fields, charge susceptibilities may also be studied in comparable nanostructures, including armchair graphene nanoribbons^74^, where field-induced changes to electronic response functions are similar to those in the dice lattice. This configuration provides a detailed representation of electronic couplings, allowing for the examination of the dice lattice’s responses to applied fields and vibrational effects. The operators $q_{i}$ and $q^{\dagger }_{i}$ annihilate and create Einstein phonons at location $i$, satisfying bosonic algebra: $[q_{i}, q^{\dagger }_{i'}] = \delta _{i,i'}$ and $[q_{i}, q_{i'}] = 0$. The phonon energy scale is $\omega _0$, and $g$ quantifies the electron-phonon interaction intensity. For computational convenience, the Hamiltonian is Fourier-transformed to wavevector space:

3 $$\begin{aligned} d^{\sigma \dagger }_{\textbf{k}} = \frac{1}{\sqrt{N}} \sum _i e^{-i \textbf{k} \cdot \textbf{R}_i} d^{\sigma \dagger }_{i}, \quad e^{\sigma \dagger }_{\textbf{k}} = \frac{1}{\sqrt{N}} \sum _i e^{-i \textbf{k} \cdot \textbf{R}_i} e^{\sigma \dagger }_{i}, \quad f^{\sigma \dagger }_{\textbf{k}} = \frac{1}{\sqrt{N}} \sum _i e^{-i \textbf{k} \cdot \textbf{R}_i} f^{\sigma \dagger }_{i}, \end{aligned}$$

where $\textbf{k} = k_x \hat{x} + k_y \hat{y}$ resides in the honeycomb’s first Brillouin zone, $N$ counts the unit cells, and $\textbf{R}_i$ locates the $i$-th cell. The resulting wavevector Hamiltonian is:

4 $$\begin{aligned} H= & -\sum _{\textbf{k},\sigma } \psi (\textbf{k}) d^{\sigma \dagger }_{\textbf{k}} e^{\sigma }_{\textbf{k}} -\sum _{\textbf{k},\sigma } \psi ^{*}(\textbf{k}) f^{\sigma \dagger }_{\textbf{k}} e^{\sigma }_{\textbf{k}}+h.c.+\sum _{\textbf{k},\sigma }(2 \mu _B B \sigma - \mu ) \left( d^{\sigma \dagger }_{\textbf{k}} d^{\sigma }_{\textbf{k}}+ e^{\sigma \dagger }_{\textbf{k}} e^{\sigma }_{\textbf{k}} + f^{\sigma \dagger }_{\textbf{k}} f^{\sigma }_{\textbf{k}} \right) \nonumber \\+ & g \sum _{\textbf{k},\textbf{q}} \left( d^{\sigma \dagger }_{\textbf{k}+\textbf{q}} d^{\sigma }_{\textbf{k}} + e^{\sigma \dagger }_{\textbf{k}+\textbf{q}} e^{\sigma }_{\textbf{k}}+f^{\sigma \dagger }_{\textbf{k}+\textbf{q}} f^{\sigma }_{\textbf{k}} \right) \left( q_{\textbf{q}} + q^{\dagger }_{-\textbf{q}} \right) + \sum _{\textbf{k}} \omega _0 q^{\dagger }_{\textbf{k}} q_{\textbf{k}}, \nonumber \\ \psi (\textbf{k})= & t \left[ 1 + 2 e^{-i \frac{\sqrt{3}}{2} k_x a} \cos (k_y a / 2) \right] . \end{aligned}$$

The function $\psi (\textbf{k})$ embodies intra-layer hopping, whereas the phonon component features $q_{\textbf{k}}$, the Einstein phonon annihilation operator in momentum space. The itinerant electron tight-binding segment of the Hamiltonian in the dice framework reads:

5 $$\begin{aligned} H_{\text {TB}} = -\sum _{\textbf{k},\sigma } \psi (\textbf{k}) d^{\sigma \dagger }_{\textbf{k}} e^{\sigma }_{\textbf{k}} -\sum _{\textbf{k},\sigma } \psi ^{*}(\textbf{k}) f^{\sigma \dagger }_{\textbf{k}} e^{\sigma }_{\textbf{k}}+h.c.+\sum _{\textbf{k},\sigma }(2 \mu _B B \sigma - \mu ) \left( d^{\sigma \dagger }_{\textbf{k}} d^{\sigma }_{\textbf{k}}+ e^{\sigma \dagger }_{\textbf{k}} e^{\sigma }_{\textbf{k}} + f^{\sigma \dagger }_{\textbf{k}} f^{\sigma }_{\textbf{k}} \right) . \end{aligned}$$

This can be reformulated as:

6 $$\begin{aligned} H_{\text {TB}} = \sum _{\textbf{k},\sigma } \Phi ^{\sigma \dagger }_{\textbf{k}} \mathcal {M}^{\sigma }(\textbf{k}) \Phi ^{\sigma }_{\textbf{k}}, \end{aligned}$$

with the basis vector $\Phi ^{\sigma \dagger }_{\textbf{k}} = (d^{\sigma \dagger }_{\textbf{k}}, e^{\sigma \dagger }_{\textbf{k}}, f^{\sigma \dagger }_{\textbf{k}})$, and the matrix $\mathcal {M}^{\sigma }(\textbf{k})$ expressed as:

7 $$\begin{aligned} \mathcal {M}^{\sigma }(\textbf{k}) = \begin{pmatrix} - \mu + 2 \mu _B B \sigma & \psi (\textbf{k}) & 0 \\ \psi ^*(\textbf{k}) & - \mu + 2 \mu _B B \sigma & \psi (\textbf{k}) \\ 0 & \psi ^*(\textbf{k}) & - \mu + 2 \mu _B B \sigma \end{pmatrix}. \end{aligned}$$

Through unitary diagonalization, the tight-binding Hamiltonian $H_{TB}$ in reciprocal space (momentum $\textbf{k}$) is projected onto the band basis, converting the matrix $\mathcal {M}^{\sigma }(\textbf{k})$ to diagonal form. The resulting band energies for the dice system under magnetic field, excluding phonon couplings, are:

8 $$\begin{aligned} \epsilon ^{\sigma }_1(\textbf{k}) = -\sqrt{2}|\psi (\textbf{k})| - \mu + 2 \sigma \mu _B B,\quad \epsilon ^{\sigma }_2(\textbf{k})= - \mu + 2 \sigma \mu _B B,\quad \epsilon ^{\sigma }_3(\textbf{k}) = \sqrt{2}|\psi (\textbf{k})| - \mu + 2 \sigma \mu _B B. \end{aligned}$$

Hence, the tight-binding Hamiltonian in the band Hilbert space becomes:

9 $$\begin{aligned} H = \sum _{l=1}^3 \sum _{\textbf{k},\sigma } \epsilon ^{\sigma }_l(\textbf{k}) g^{\sigma \dagger }_{l,\textbf{k}} g^{\sigma }_{l,\textbf{k}}, \end{aligned}$$

where $l = 1, 2, 3$ labels the bands, and $g^{\sigma \dagger }_{l,\textbf{k}}$ generates an electron in band $l$ with momentum $\textbf{k}$ and spin $\sigma$. The band-space fermionic operators connect to the sublattice-space ones by:

10 $$\begin{aligned} \begin{pmatrix} g^{\sigma \dagger }_{1,\textbf{k}} \\ g^{\sigma \dagger }_{2,\textbf{k}} \\ g^{\sigma \dagger }_{3,\textbf{k}} \end{pmatrix} = \begin{pmatrix} u_{1,A}(\textbf{k}) & u_{1,B}(\textbf{k}) & u_{1,C}(\textbf{k}) \\ u_{2,A}(\textbf{k}) & u_{2,B}(\textbf{k}) & u_{2,C}(\textbf{k}) \\ u_{3,A}(\textbf{k}) & u_{3,B}(\textbf{k}) & u_{3,C}(\textbf{k}) \\ \end{pmatrix} \begin{pmatrix} d^{\sigma \dagger }_{\textbf{k}} \\ e^{\sigma \dagger }_{\textbf{k}} \\ f^{\sigma \dagger }_{\textbf{k}} \end{pmatrix}, \end{aligned}$$

where $u_{l,\gamma }(\textbf{k})$ (for $l = 1, 2, 3$, $\gamma = A, B, C$) are the complex unitary transformation coefficients, omitted here for conciseness.

## Green’s functions

Green’s functions serve as a fundamental tool in condensed matter physics for tackling intricate issues such as electron correlations, phonon dynamics, and metallic resistivity^75^. Compared to alternative techniques, they offer a unique strategy for simulating and solving problems in solids, particularly those involving complex boundaries or intense confinements. Their versatility in addressing diverse phenomena makes them indispensable in a physicist’s arsenal. Furthermore, in the Hückel tight-binding context, Green’s functions facilitate the computation of matrix determinants and inverses pertinent to quantum chemistry and solid-state studies^76,77^.

### Non-interacting green’s functions

The components of the bare single-particle Green’s function are derived from its operator-based definition, adhering to fermionic anticommutation rules. Due to the three-atom unit cell, the Green’s function forms a $3 \times 3$ matrix in sublattice space. The Matsubara components are:

11 $$\begin{aligned} \mathcal {G}^{\sigma }_{\gamma \delta }(\textbf{k}, \tau )= & -\langle T_\tau \gamma ^{\sigma }_{\textbf{k}}(\tau ) \delta ^{\sigma \dagger }_{\textbf{k}}(0) \rangle ,\nonumber \\ \mathcal {G}^{\sigma }_{\gamma \delta }(\textbf{k}, i \omega _p)= & \int _0^{1/(k_B T)} e^{i \omega _p \tau } \mathcal {G}^{\sigma }_{\gamma \delta }(\textbf{k}, \tau ) \, d\tau , \qquad \omega _p=(2p+1)\pi k_{B}T, \end{aligned}$$

with $\{\gamma ,\delta \}=\{d,e,f\}$ and $\omega _p$ as the fermionic Matsubara frequencies. Here, $\tau$ represents imaginary time, and $T$ is the system’s thermal equilibrium temperature. For the dice system, the unit cell’s three atoms and the Hamiltonian’s symmetry yield nine single-particle Green’s function elements. From Eqs. (10,11), the Fourier-transformed bare Green’s function elements in sublattice space are:

12 $$\begin{aligned} \mathcal {G}^{(0)\sigma }_{\gamma \delta }(\textbf{k}, i \omega _p) = \sum _{l=1}^3 \frac{u_{l,\gamma }(\textbf{k}) u^*_{l,\delta }(\textbf{k})}{i \omega _p - \epsilon ^{\sigma }_l(\textbf{k})}, \end{aligned}$$

where $u_{l,\gamma }$ are the unitary elements from Eq. (10).

### Interacting green’s functions

To incorporate phonon effects, we evaluate the electron-phonon term in the Holstein Hamiltonian from Eq. (4), expressed as:

13 $$\begin{aligned} H_{\text {e-ph}} = g \sum _{\textbf{k},\textbf{q}} \left( d^{\sigma \dagger }_{\textbf{k}+\textbf{q}} d^{\sigma }_{\textbf{k}} + e^{\sigma \dagger }_{\textbf{k}+\textbf{q}} e^{\sigma }_{\textbf{k}}+f^{\sigma \dagger }_{\textbf{k}+\textbf{q}} f^{\sigma }_{\textbf{k}} \right) \left( q_{\textbf{q}} + q^{\dagger }_{-\textbf{q}} \right) . \end{aligned}$$

This interaction component modifies the energy dispersion and functions of the Green.Dyson’s relation yields the dressed matrix by using Matsubara perturbation theory for the spin-resolved interacting Green’s function matrix.Migdal’s approximation^78^ confirms that the huge ion-electron mass ratio justifies ignoring vertex diagrams in the electron self-energy, given the enormous difference in phonon and electron energy scales. From Dyson’s equation, the clothed Green’s function is obtained, including electron-phonon effects into the band analysis. By accurately evaluating vibrational effects on the system, our approach expands on our understanding of electron-phonon synergy in the dice framework.

14 $$\begin{aligned} \mathbf {\mathcal {G}}^{\sigma }(\textbf{k}, i \omega _p)= & \mathbf {\mathcal {G}}^{(0)\sigma }(\textbf{k}, i \omega _p) + \mathbf {\mathcal {G}}^{(0)\sigma }(\textbf{k}, i \omega _p) \mathbf {\Pi }^{\sigma }(\textbf{k}, i \omega _p) \mathbf {\mathcal {G}}^{\sigma }(\textbf{k}, i \omega _p), \end{aligned}$$

where $\mathbf {\mathcal {G}}^{(0)\sigma }$ and $\mathbf {\Pi }^{\sigma }$ are $3 \times 3$ sublattice matrices. The self-energy elements arise from second-order perturbation:

15 $$\begin{aligned} \Pi ^{\sigma }_{\gamma ,\delta }(\textbf{k}, i \omega _p) = -k_B T \sum _{\textbf{q},r} g^2 D^{(0)}(\textbf{q}, i \Lambda _r) \mathcal {G}^{(0)\sigma }_{\gamma ,\delta }(\textbf{k} - \textbf{q}, i \omega _p - i \Lambda _r), \end{aligned}$$

with $\Lambda _r = 2 r \pi k_B T$ as bosonic Matsubara frequencies, and the phonon Green’s function:

16 $$\begin{aligned} D^{(0)}(\textbf{q}, i \Lambda _r) = \int _0^{1/(k_B T)} d\tau e^{i \Lambda _r \tau } \langle T_\tau (q_{\textbf{q}}(\tau ) + q^{\dagger }_{-\textbf{q}}(\tau ))(q_{\textbf{q}}(0) + q^{\dagger }_{-\textbf{q}}(0)) \rangle = \frac{2 \omega _0}{(i \Lambda _r)^2 - \omega _0^2}, \end{aligned}$$

disregarding screening^79^. Inserting Eq. (12) and Eq. (16) into Eq. (15) gives the self-energy:

17 $$\begin{aligned} \Pi ^{\sigma }_{\gamma ,\delta }(\textbf{k}, i \omega _p)&= \frac{g^2}{2 N} \sum _{\textbf{q},l=1}^3 u_{l,\gamma }(\textbf{k} - \textbf{q}) u^*_{l,\delta }(\textbf{k} - \textbf{q}) \nonumber \\&\quad \times \left( \frac{n_B(\omega _0) + n_F(\epsilon ^{\sigma }_l(\textbf{k} - \textbf{q}))}{i \omega _p - \epsilon ^{\sigma }_l(\textbf{k} - \textbf{q}) + \omega _0} + \frac{n_B(\omega _0) + 1 - n_F(\epsilon ^{\sigma }_l(\textbf{k} - \textbf{q}))}{i \omega _p - \epsilon ^{\sigma }_l(\textbf{k} - \textbf{q}) - \omega _0} \right) , \end{aligned}$$

where $n_F(x) = \frac{1}{e^{x/(k_B T)} + 1}$ and $n_B(\omega _0) = \frac{1}{e^{\omega _0/(k_B T)} - 1}$ denote Fermi-Dirac and Bose-Einstein functions.

In band space, the bare Green’s function is diagonal:

18 $$\begin{aligned} \mathcal {G}^{(0)\sigma }_{ll}(\textbf{k}, i \omega _p) = \frac{1}{i \omega _p - \epsilon ^{\sigma }_l(\textbf{k})}, \end{aligned}$$

and the band-projected self-energy is:

19 $$\begin{aligned} \Pi ^{\sigma }_{ll}(\textbf{k}, i \omega _p) = \sum _{\gamma ,\delta } u^*_{l,\gamma }(\textbf{k}) u_{l,\delta }(\textbf{k}) \Pi ^{\sigma }_{\gamma \delta }(\textbf{k}, i \omega _p). \end{aligned}$$

The dressed band Green’s function reads:

20 $$\begin{aligned} \mathcal {G}^{\sigma }_{ll}(\textbf{k}, i \omega _p) = \frac{1}{i \omega _p - \epsilon ^{\sigma }_l(\textbf{k}) - \Pi ^{\sigma }_{l,l}(\textbf{k}, i \omega _p)}. \end{aligned}$$

The chemical potential $\mu$ derives from electron density $n_e$:

21 $$\begin{aligned} n_e = \frac{1}{3 N} \sum _{\textbf{k},l,\sigma } \frac{1}{e^{\epsilon ^{\sigma }_l(\textbf{k})/(k_B T)} + 1}, \end{aligned}$$

numerically resolved for specified $n_e$ and temperature. The electron-phonon-inclusive density of states (DOS) for the dice lattice is:

22 $$\begin{aligned} D(E) = -\frac{1}{3 \pi N} \sum _{\sigma ,\textbf{k},l} \text {Im} \mathcal {G}^{\sigma }_{ll}(\textbf{k}, E + i 0^+), \end{aligned}$$

The integration spans the Brillouin zone, with DOS maxima at band boundaries signifying van Hove singularities where electronic density peaks. Linear response formalism is utilized to derive transport coefficients, evaluating thermal and thermoelectric responses of the dice system under mild gradients in electric field and temperature. These coefficients, $\mathcal {L}_{11}, \mathcal {L}_{12}, \mathcal {L}_{22}$, connect charge and heat currents to electric potential gradient $\nabla V$ and temperature gradient $\nabla T$ as driving fields^80^:

23 $$\begin{aligned} & \textbf{J}^{e}=\mathcal {L}_{11}(-\nabla V)+\mathcal {L}_{12}(-\nabla T),\nonumber \\ & \textbf{J}^{Q}=\mathcal {L}_{21}(-\nabla V)+\mathcal {L}_{22}(-\nabla T), \end{aligned}$$

where $\textbf{J}^{e}$ ($\textbf{J}^{Q}$) is the electric (heat) current density. The $L_{ab}$ (for $a,b=1,2$) arise from correlations of electric and thermal current operators:

24 $$\begin{aligned} \mathcal {L}_{11}= & \frac{e^{2}k_{B}T}{3N}\sum _{\textbf{k},l}\sum _{\sigma }\Big (\frac{\partial \epsilon ^{\sigma }_l(\textbf{k})}{\partial k_{x}}\Big )^{2}\int _{-\infty }^{\infty }\frac{dE}{2\pi } \frac{-\partial n_{F}(E)}{\partial E} \Big (-2\text {Im} \mathcal {G}^{\sigma }_{ll}(\textbf{k}, E + i 0^+)\Big )^{2},\nonumber \\ \mathcal {L}_{12}= & \frac{e k_{B}T}{3N}\sum _{\textbf{k},l}\sum _{\sigma }\Big (\frac{\partial \epsilon ^{\sigma }_l(\textbf{k})}{\partial k_{x}}\Big )^{2}\int _{-\infty }^{\infty }\frac{dE}{2\pi }E \frac{-\partial n_{F}(E)}{\partial E} \Big (-2\text {Im} \mathcal {G}^{\sigma }_{ll}(\textbf{k}, E + i 0^+)\Big )^{2},\nonumber \\ \mathcal {L}_{22}= & \frac{e k_{B}T}{3N}\sum _{\textbf{k},l}\sum _{\sigma }\Big (\frac{\partial \epsilon ^{\sigma }_l(\textbf{k})}{\partial k_{x}}\Big )^{2}\int _{-\infty }^{\infty }\frac{dE}{2\pi }E^{2} \frac{-\partial n_{F}(E)}{\partial E} \Big (-2\text {Im} \mathcal {G}^{\sigma }_{ll}(\textbf{k}, E + i 0^+)\Big )^{2}. \end{aligned}$$

These coefficients are computed numerically via wavevector summation over the first Brillouin zone, incorporating the electronic Green’s function into Eq. (24). From these, the dice lattice’s transport and thermoelectric attributes are derived. The dc electrical conductivity along $x$ with electron-phonon effects relates to $\mathcal {L}_{11}$ as^80^:

25 $$\begin{aligned} \sigma (T)=\frac{1}{T}\mathcal {L}_{11}. \end{aligned}$$

In open-circuit conditions ($\textbf{J}^{e}=0$), heat flow responds to temperature gradients: $\textbf{J}^{Q}=K \nabla T$, with thermal conductivity $K$ from transport coefficients^79^:

26 $$\begin{aligned} K= & \frac{1}{T^2}(\mathcal {L}_{22}-\frac{\mathcal {L}_{12}^{2}}{\mathcal {L}_{11}}). \end{aligned}$$

Subsequent focus is on the system’s thermoelectric traits. The thermopower, or Seebeck coefficient, quantifies the voltage induced per unit temperature difference across the material: $S=|\nabla V|/|\nabla T|$, with $\nabla V$ as the spatial voltage drop^79^. Within linear response, it connects to coefficients via:

27 $$\begin{aligned} S=-\frac{1}{T}\frac{\mathcal {L}_{12}}{\mathcal {L}_{11}}. \end{aligned}$$

This thermopower elucidates electricity generation from thermal gradients, with its sign revealing dominant heat carriers in structures like bilayer graphene. Another key thermoelectric metric, the figure of merit $ZT$, derives from coefficients as^80^:

28 $$\begin{aligned} ZT=\frac{\sigma S^{2}}{K}T. \end{aligned}$$

Per this relation, efficient thermoelectrics demand low thermal conductivity alongside elevated thermopower and electrical conductivity. Additionally, coefficients yield power factor $PF$ and Lorenz number $L$. The power factor follows from coefficients as^81^:

29 $$\begin{aligned} PF=\frac{\mathcal {L}^{2}_{12}}{T^{2}\mathcal {L}_{11}}. \end{aligned}$$

The Wiedemann-Franz relation links electrical and thermal conductivities via the Lorenz number $L$. As carriers (electrons in n-type or holes in p-type) convey both charge and heat, $K$ is often inferred from measured $\sigma$ using this law, assuming a non-interacting electron gas. The Lorenz number, the thermal-to-electrical conductivity ratio, is:

30 $$\begin{aligned} L=\frac{K}{\sigma T}=\frac{\mathcal {L}_{11}\mathcal {L}_{22}-\mathcal {L}^{2}_{12}}{T^{2}\mathcal {L}^{2}_{11}}. \end{aligned}$$

## Results and analysis

In this section, we delineate the computational findings pertaining to the electronic, transport, and thermoelectric attributes of a two-dimensional dice lattice, modulated by an applied perpendicular magnetic field and electron-phonon interactions as modeled by the Holstein framework. Representative outcomes are showcased for key electronic and transport metrics, including the density of states, electrical conductivity, thermal conductivity, and Seebeck coefficient. In addition, we investigate the temperature changes of several of the lattice’s thermoelectric properties, such as power factor, Lorenz number, and figure of merit. Furthermore, under electron-Holstein phonon interactions, the effect of electron doping on the thermal profiles of electrical and thermal conductivities is investigated inside the dice lattice. The role of the magnetic field in shaping the chemical potential profiles of the Seebeck coefficient and thermal conductivity is also scrutinized. As elaborated in the preceding section, these characteristics are derived through the application of Green’s function methodology in the Holstein paradigm, wherein the dressed Green’s functions are computed via a one-loop perturbation for the self-energy.

The band-projected self-energy $\Sigma _{mm}(\vec {k}, i\nu _n)$ is derived through numerical evaluation of the self-energy components in the sublattice framework as per Eq. (17), followed by their transformation to the band formalism via Eq. (19). Subsequently, the dressed Green’s function is computed according to Eq. (20). The density of states is then ascertained from Eq. (22) employing the interacting Green’s function $\mathcal {G}^{\sigma }_{mm}(\vec {k}, E)$. The transport coefficients are found by performing numerical quadrature over the wave vectors in the first Brillouin zone and entering the interacting electronic Green’s function for the dice lattice into Eq. (24). Using Eqs. (26), (27), (28), (29), and (30), respectively, these coefficient values are then used to produce the corresponding numerical results for thermal conductivity, Seebeck coefficient, figure of merit, and power factor of the dice lattice.

**Fig. 2:**
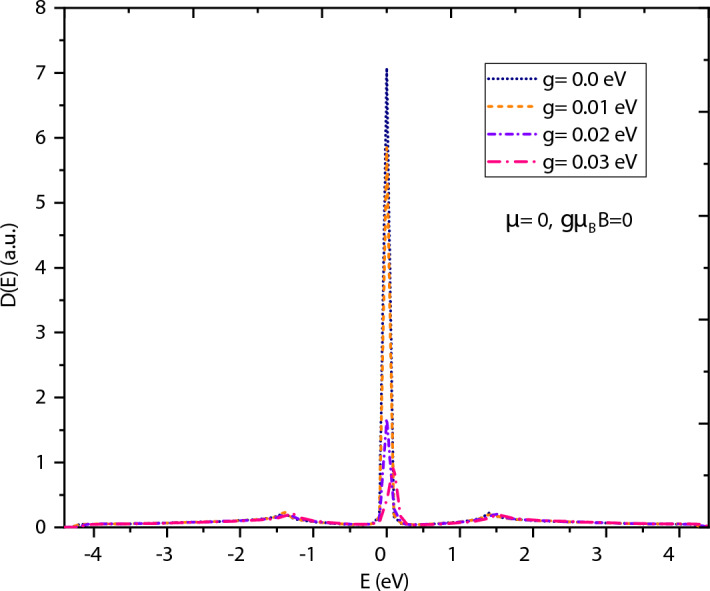
The electronic density of states (DOS) as a function of energy in an undoped dice lattice, influenced by differing electron-phonon coupling parameters *g*, in the absence of an external magnetic field.

Figure 2 illustrates the energy-dependent density of states (DOS), labeled as $D(E)$, for an undoped dice lattice under varying electron-phonon coupling strengths $g$, with no external magnetic field applied. The overall shape of the DOS is markedly altered by the intensity of these electron-phonon interactions. For each coupling value, a prominent peak appears at zero energy, originating from the dispersionless flat band inherent in the low-energy excitation spectrum of the dice lattice. In the absence of coupling ($g = 0$), the system exhibits metallic behavior, characterized by a finite DOS at the Fermi level ($D(E = \mu = 0) \ne 0$), which facilitates electron conduction at zero temperature. As the coupling parameter $g$ increases, the amplitude of this zero-energy peak diminishes, reflecting a reduction in the DOS at the Fermi level; This effect results from polaronic dressing’s renormalization of electronic states, which causes electrons to become quasiparticles attached to lattice distortions. This effectively widens the flat band and reduces its contribution to low-energy states, which lessens the material’s metallic qualities. Stronger coupling increases scattering and self-energy adjustments that blur out sharp features in the DOS, which is consistent with perturbation theory predictions in the Holstein model. Furthermore, the mirror-like profile of the DOS is maintained by the inherent particle-hole symmetry of the Hamiltonian, guaranteeing $D(E) = D(-E)$ with respect to $E \approx 0$. This is a result of the model’s chiral framework’s balanced handling of electron and hole excitations.

**Fig. 3:**
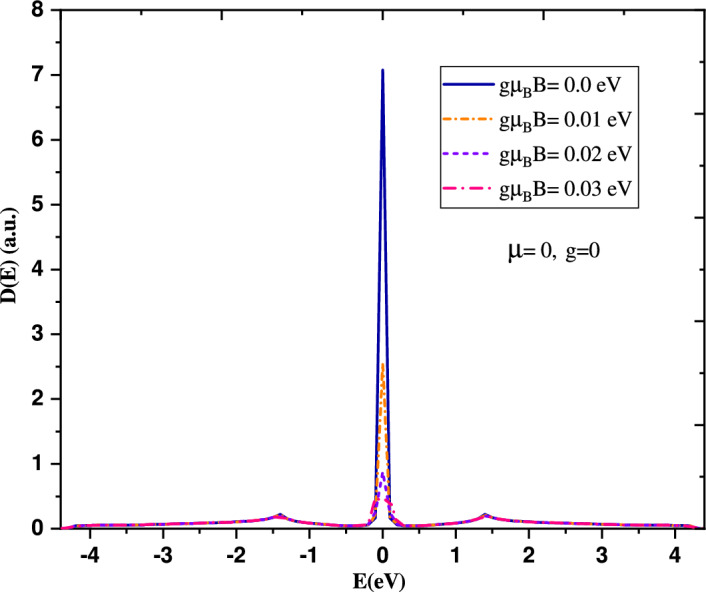
The electronic density of states (DOS) as a function of energy in an undoped dice lattice, modulated by varying magnetic field strengths $g\mu _B B$, in the absence of electron-phonon coupling.

Figure 3 displays the energy-dependent density of states *D*(*E*) of an undoped dice lattice at fixed temperature $k_B T / t = 0.03$, in the absence of electron-phonon coupling ($g = 0$), for magnetic fields ranging from $g \mu _B B / t = 0$ to 0.03. As the magnetic field increases, the density of states at zero energy decreases steadily, reaching its minimum at the highest field strength. In zero field the system is metallic, with a pronounced peak at the Fermi level that guarantees finite conductivity even at low temperatures. Application of the field splits the degenerate flat band through the Zeeman term, suppressing its contribution at $E = 0$ and opening a small gap proportional to the field strength. Consequently, the lattice gradually acquires semimetallic to weakly insulating character. This behavior arises from the breaking of chiral symmetry in the pseudospin-1 Dirac system and demonstrates that a perpendicular magnetic field provides a powerful handle for tuning the electronic structure of dice lattices, with direct implications for designing spintronic devices (via controllable spin-polarized transport) and quantum-information platforms that exploit field-induced manipulation of flat-band localization and Dirac-fermion dynamics To initiate the analysis of thermal transport properties in the dice lattice, we first examine the transport processes governed by the external magnetic field, electron-phonon coupling strength, and variations in chemical potential.

**Fig. 4:**
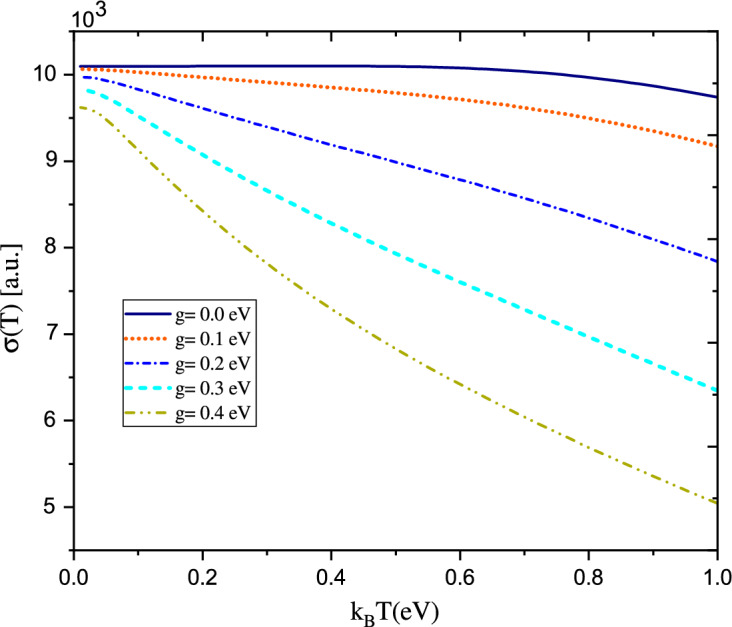
Electrical conductivity in an undoped dice lattice plotted against temperature $k_B T$, examined for multiple electron-phonon coupling strengths such as $g = 0.0$ eV, 0.1 eV, 0.2 eV, 0.3 eV, and 0.4 eV, in the absence of a magnetic field.

Figure 4 shows the temperature dependence of the electrical conductivity $\sigma$ in an undoped dice lattice for electron-phonon coupling strengths $g = 0.0$, 0.1, 0.2, 0.3, and 0.4 eV. For all values of *g*, the conductivity remains finite in the zero-temperature limit. This behavior stems from the persistent nonzero density of states at the chemical potential ($\mu = 0$), as previously illustrated in Fig. 2. Consequently, the system exhibits metallic character at half-filling, allowing charge transport even without thermal excitation due to available states at the Fermi level. As *g* increases, the zero-energy DOS decreases owing to self-energy corrections that broaden the flat band through polaron formation; hence the low-temperature limit of $\sigma$ is progressively reduced. At any fixed temperature, stronger electron-phonon coupling enhances scattering of charge carriers by lattice vibrations, since more electrons become coupled to localized phonon modes, thereby impeding momentum relaxation and lowering $\sigma$. Moreover, with rising temperature the phonon population increases according to Bose-Einstein statistics, intensifying electron-phonon scattering events and causing a continuous decline in conductivity. This temperature-induced suppression is typical of metallic systems at half-filling ($\mu = 0$), where the Fermi surface remains populated and thermal smearing amplifies resistive losses in accordance with Matthiessen’s rule for multiple scattering mechanisms.

**Fig. 5:**
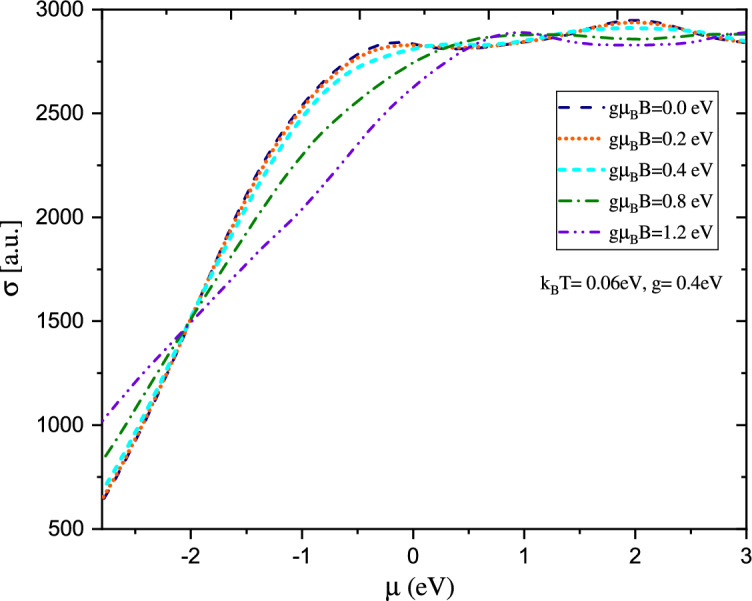
Electrical conductivity in a doped dice lattice plotted as a function of chemical potential $\mu$, under various magnetic field strengths including $g\mu _B B = 0.0$ eV, 0.2 eV, 0.4 eV, 0.8 eV, and 1.2 eV, at a constant temperature of $k_B T = 0.06$ eV, with electron-phonon coupling fixed at $g = 0.4$ eV.

Figure 5 presents the electrical conductivity $\sigma$ of the dice lattice as a function of chemical potential $\mu$ under various magnetic field strengths, at fixed temperature $k_B T = 0.06$ eV and electron-phonon coupling $g = 0.4$ eV. For all magnetic fields, $\sigma$ increases monotonically in the p-type doping regime ($\mu < 0$) owing to the doping-induced increase in charge carrier concentration, which shifts the Fermi level and enables more efficient interband transitions, thereby enhancing transport efficiency. For positive chemical potentials ($\mu > 0$), however, this monotonic rise gives way to a non-monotonic dependence because enhanced carrier scattering at higher densities counteracts the benefit of increased conduction-band population. Increasing the magnetic field raises $\sigma$ at fixed $\mu < -2.0$ eV, as Zeeman splitting promotes spin polarization and suppresses spin-dependent scattering. In contrast, within the interval $-2.0$ eV $< \mu < 0$, stronger fields reduce $\sigma$ by lowering the density of states near the Fermi energy – consistent with the DOS behavior shown earlier – thereby restricting the available conduction channels in this pseudospin-1 system.

**Fig. 6:**
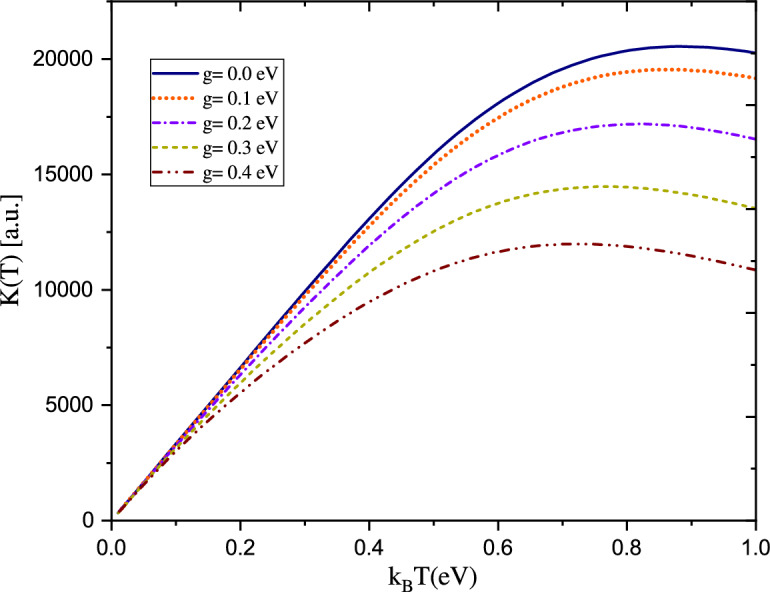
Thermal conductivity in an undoped dice lattice illustrated as a function of temperature $k_B T$, across various electron-phonon coupling strengths such as $g = 0.0$ eV, 0.1 eV, 0.2 eV, 0.3 eV, and 0.4 eV, under zero magnetic field conditions.

Figure 6 illustrates the thermal conductivity as a function of temperature for different electron-phonon coupling strengths $g$, under zero magnetic field. As temperature rises, charge carriers gain greater thermal energy, facilitating excitations from the valence band to the conduction band; this boosts thermal conductivity by increasing heat-transporting quasiparticles (electrons and phonons), consistent with the kinetic theory where $\kappa \propto C_v v l$ (with $C_v$ as specific heat, $v$ as velocity, and $l$ as mean free path). The trend rises to a maximum, then declines due to intensified carrier scattering from higher conduction band population–via Umklapp phonon-phonon or carrier-carrier processes common in Dirac-like dispersions–reducing the mean free path. Stronger coupling (higher $g$) further lowers $\kappa$ through increased electron-phonon scattering, shortening quasiparticle lifetimes and impeding heat transfer. At low temperatures below  0.1 eV (corresponding to $k_B T \approx 0.1 t$, where $t$ is the hopping parameter), $\kappa$ remains largely insensitive to $g$ variations, with curves converging, as phonon occupations follow Bose-Einstein statistics and are exponentially suppressed at low $T$, minimizing coupling effects until thermal activation becomes significant.

**Fig. 7:**
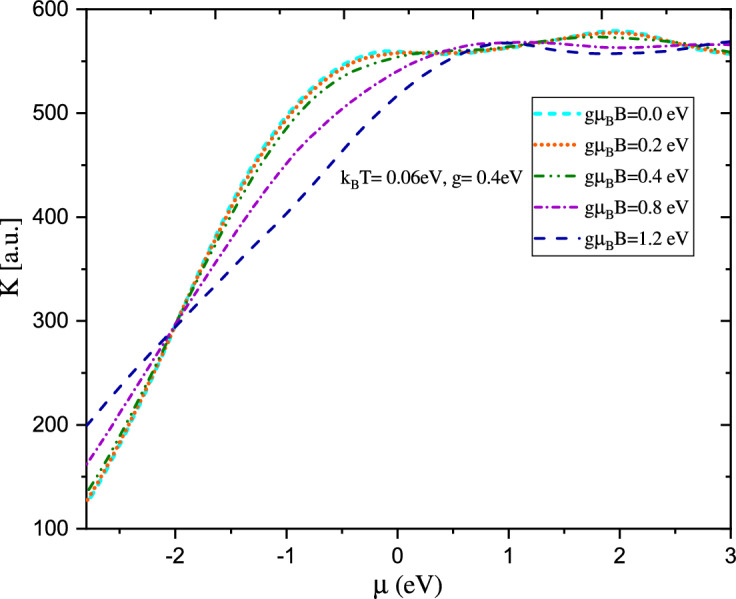
Thermal conductivity in a doped dice lattice illustrated as a function of chemical potential $\mu$, across various magnetic field strengths including $g\mu _B B = 0.0$ eV, 0.2 eV, 0.4 eV, 0.8 eV, and 1.2 eV, at a constant temperature of $k_B T = 0.06$ eV, with electron-phonon coupling fixed at $g = 0.4$ eV.

Figure 7 shows the dependence of thermal conductivity $K$ on chemical potential for a doped dice lattice, evaluated under multiple magnetic field strengths, with fixed parameters of $k_B T = 0.06$ eV and electron-phonon coupling $g = 0.4$ eV. Notably, a monotonic increase in electronic thermal conductivity occurs throughout the p-type doping range ($\mu < 0$ eV) for all magnetic fields, driven by doping that elevates carrier concentration; this enables more effective heat transfer via enhanced phonon and electron-mediated processes. The system’s electron density increases when the chemical potential rises, which encourages faster transitions between quantum energy levels and improves heat propagation. However, the behavior becomes non-monotonic for $\mu > 0$ eV due to enhanced carrier scattering (e.g., more electron-electron collisions at higher densities), which shortens the mean free path and reduces thermal efficiency despite surplus charge carriers. In the profoundly p-doped zone ($\mu < -2.0$ eV), stronger magnetic fields increase $K$ through Zeeman splitting at constant chemical potentials. This improves heat conduction by aligning spin configurations, extending quasiparticle lifetimes, and reducing spin-flip scattering. On the other hand, as Fig. 7 illustrates, increasing the magnetic field lowers (“K”) in the intermediate p-type range of $-2.0$ eV $< \mu < 0.0$ eV, due to suppression of the density of states near the Fermi energy, which limits the number of states available for thermal transport and mimics gap-opening effects in pseudospin-1 Dirac systems.

**Fig. 8:**
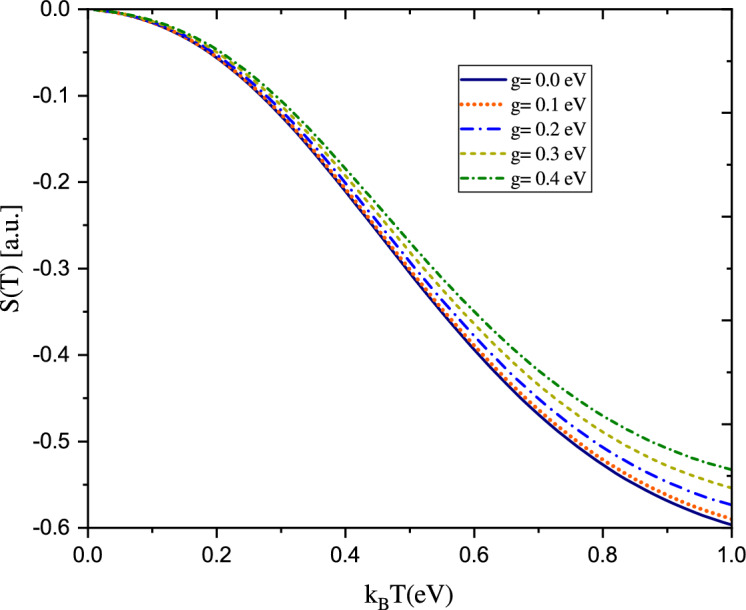
Seebeck coefficient in an undoped dice lattice depicted as a function of temperature $k_B T$, analyzed across various electron-phonon coupling strengths including $g = 0.0$ eV, 0.1 eV, 0.2 eV, 0.3 eV, and 0.4 eV, under zero magnetic field conditions.

We have also studied the temperature dependence of thermoelectric properties of dice lattice due to the electron-phonon coupling effects. The Seebeck coefficient of undoped monolayer dice lattice as a function of temperature for various coupling constants *g* in the absence of magnetic field is demonstrated in Fig. 8. It is well-established that the sign of the Seebeck coefficient serves as a criterion for determining the predominant carrier type. A positive (negative) Seebeck coefficient indicates that the charge and heat are predominantly carried holes (electrons)^82^. In this figure, the negative values of this coefficient indicate the predominance of electrons in the heat flow process within the dice lattice. Essentially, this signifies that the dice monolayer behaves as a n-type semiconductor; so that with the application of electron-phonon coupling, electrons serve as the primary agents for heat transfer. The Seebeck coefficient monotonically reduces with temperature for each value of *g*. Moreover it is observed that absolute value of *S* decreases with enahncement of *g*. Such issue implies that the increase of electron-phonon coupling strength leads to decrease the number of charge carriers participating in the current. At very low temperatures, Seebeck coefficient is less *g* dependent so that the variations of electron-phonon coupling has no considerable effect on Seebeck coefficient amounts in this temperature region.

**Fig. 9:**
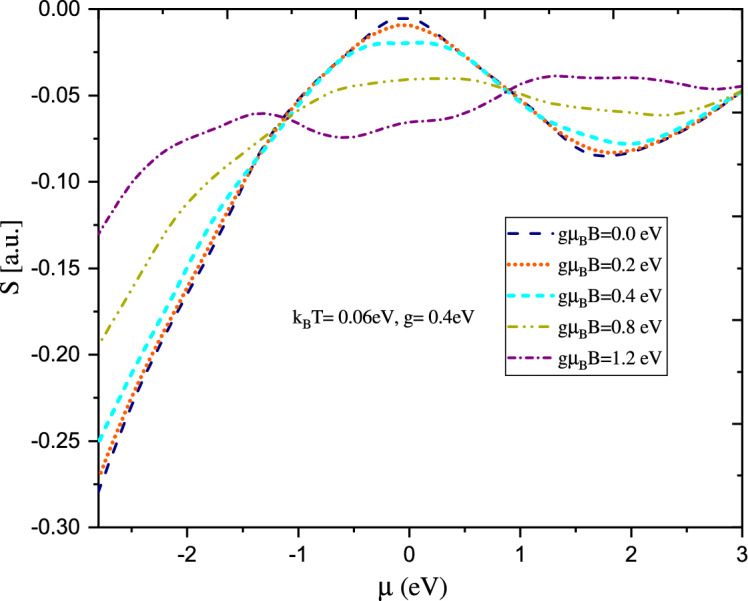
Seebeck coefficient in a doped dice lattice depicted as a function of chemical potential $\mu$, across various magnetic field strengths including $g\mu _B B = 0.0$ eV, 0.2 eV, 0.4 eV, 0.8 eV, and 1.2 eV, at a constant temperature of $k_B T = 0.06$ eV, with electron-phonon coupling fixed at $g = 0.4$ eV.

Figure 9 displays the Seebeck coefficient *S* as a function of chemical potential $\mu$. The results are shown under various magnetic field strengths. The temperature is fixed at $k_B T = 0.06$ eV and the electron-phonon coupling at $g = 0.4$ eV. As established by Furukawa, the sign of *S* indicates the dominant charge carrier species. Positive values correspond to hole-mediated transport. Negative values signify electron-dominated charge and heat flow. The coefficient remains negative over the entire range of $\mu$. This confirms that electrons govern both electrical and thermal transport in the dice lattice. In the regions $-3$ eV $< \mu < -1$ eV and 1 eV $< \mu < 3$ eV, increasing the magnetic field reduces |*S*| . This is consistent with Zeeman-induced symmetrization of electron-hole contributions. Such symmetrization weakens the thermoelectric asymmetry. In contrast, within the central interval $-1$ eV $< \mu < 1$ eV, stronger fields enhance |*S*| . This arises from intensified carrier entropy gradients. These gradients result from field-induced suppression of states near the Fermi level in the flat band. These results demonstrate that tuning the electron-phonon coupling *g* and magnetic field strength offers an effective strategy to optimize thermoelectric performance.

**Fig. 10:**
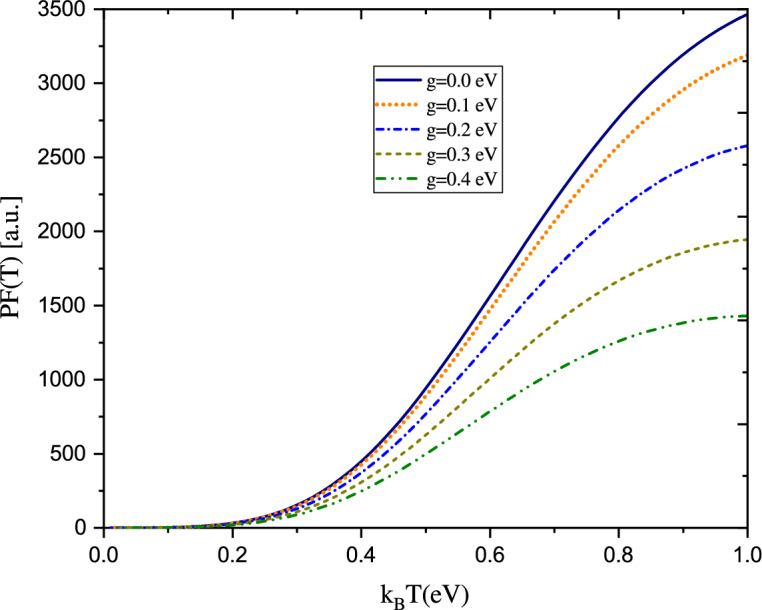
Power factor in an undoped dice lattice plotted as a function of temperature $k_B T$, examined for various electron-phonon coupling strengths such as $g = 0.0$ eV, 0.1 eV, 0.2 eV, 0.3 eV, and 0.4 eV, in the absence of a magnetic field.

Figure 10shows the power factor of an undoped dice lattice as a function of temperature $k_B T$. Different electron-phonon coupling strengths *g* are considered. For $k_B T > 0.2$ eV, the power factor increases with temperature. This occurs due to thermal excitation of additional charge carriers into the conduction band. The increase simultaneously enhances both electrical conductivity and the Seebeck coefficient. Consequently, the power factor $PF = \sigma S^2$ rises. This behavior aligns with Mott’s formula for diffusive thermopower in metallic systems. More quasiparticles improve thermoelectric conversion efficiency. Increasing *g* suppresses the power factor. It raises the effective carrier mass through polaronic effects. This hinders promotion of electrons from the valence to the conduction band. As a result, both conductivity and thermopower decrease. Below $k_B T = 0.2$ eV, the power factor remains very low. It is nearly independent of *g*. In this regime thermal activation is minimal. Phonon populations are frozen out according to Bose-Einstein statistics. Electron-phonon interactions exert negligible influence until energies exceed the characteristic phonon scale.

**Fig. 11:**
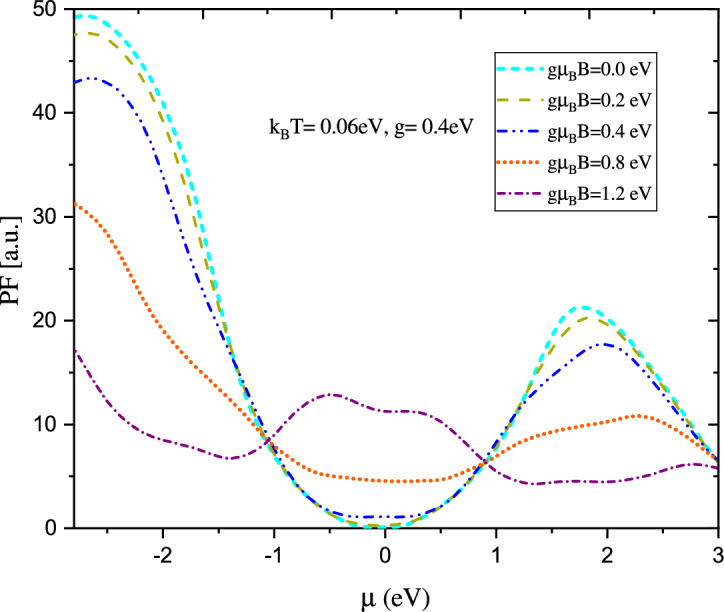
Power factor in a doped dice lattice illustrated as a function of chemical potential $\mu$, under various magnetic field strengths such as $g\mu _B B = 0.0$ eV, 0.2 eV, 0.4 eV, 0.8 eV, and 1.2 eV, at a fixed temperature of $k_B T = 0.06$ eV, with the electron-phonon coupling strength set to $g = 0.4$ eV.

Figure 11 looks at how the power factor in the dice lattice depends on chemical potential under different magnetic field strengths. The temperature is set at $k_B T = 0.06$ eV and electron-phonon coupling at $g = 0.4$ eV. In the p-type doping range (-3 eV < $\mu$ < -1 eV) and n-type region (1 eV < $\mu$ < 3 eV), the power factor drops as magnetic field intensity rises. This is probably because Zeeman splitting symmetrizes the band structure and lessens the asymmetry needed for strong thermoelectric response. By contrast, near half-filling (-1 eV < $\mu$ < 1 eV), the power factor increases with stronger fields. This indicates that magnetic effects improve carrier selectivity or curb dissipative paths around the Dirac point, optimizing PF. So, boosting $g \mu _B B$ in this central range can enhance the dice lattice’s overall thermoelectric performance. One key feature in Fig. 11 is the gradual drop in PF with chemical potential in the deeply p-type area ($\mu$ < -1.5 eV). This is due to carrier addition reaching saturation. There’s also a clear peak in the power factor around $\mu \approx 2.0$ eV for fields of $g \mu _B B = 0.0$ eV, 0.2 eV, and 0.4 eV. This stems from the Fermi level lining up well with high-DOS areas in the conduction band, where conductivity and thermopower combine to maximize energy conversion.

**Fig. 12:**
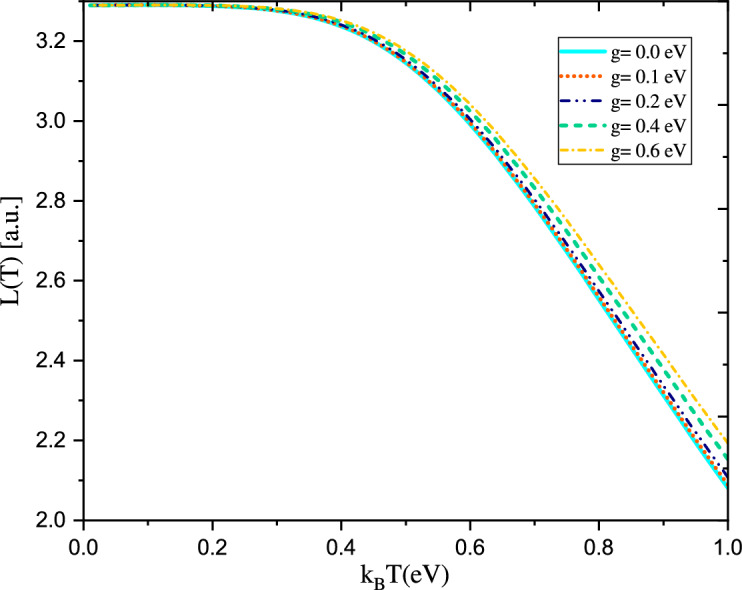
Lorenz number in an undoped dice lattice illustrated as a function of temperature $k_B T$, across various electron-phonon coupling strengths such as $g = 0.0$ eV, 0.1 eV, 0.2 eV, 0.4 eV, and 0.6 eV, in the absence of a magnetic field.

**Fig. 13:**
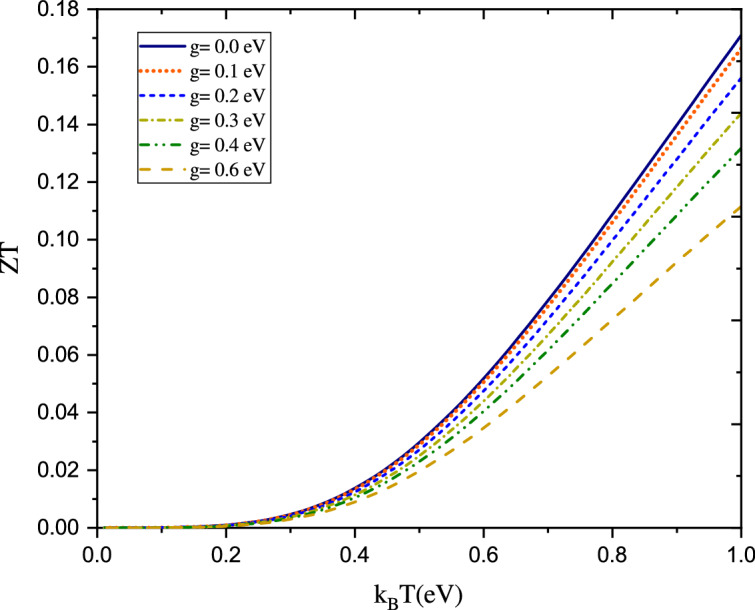
The temperature dependence of the thermoelectric figure of merit $ZT$ for an undoped dice lattice (half-filling) under various electron-phonon coupling strengths $g$ in the absence of magnetic field.

Another thermoelectric parameter under investigation is the Lorentz constant. Changes of this feature in terms of temperature for various electron-phonon coupling constants in the absence of magnetic field are shown in Fig. 12. This constant is directly correlated with thermal conductivity and inversely related to the product of electrical conductivity and temperature. For temperatures below characteristic value 0.3 eV, Lorenz number presents a constant value for all values of *g*. Such constant value for *L* is independent of electron-phonon coupling constant according to Fig. 12. Upon more increase of temperature above 0.3 eV, Lorenz number decreases with temperature for each value of *g*. In addition, at fixed temperature in the region $k_{B}T>$0.4 eV, the enhancement of coupling constant increases Lorenz number. Figure 13 depicts the temperature-dependent evolution of the figure of merit $ZT$ for an undoped dice lattice (at half-filling) across various electron-phonon coupling strengths $g$, in the absence of a magnetic field. Defined as $ZT = \sigma S^2 T / \kappa$, this dimensionless parameter quantifies a material’s thermoelectric efficiency, balancing electrical power generation against thermal losses, with higher values indicating superior conversion of heat to electricity. As temperature rises, charge carriers acquire sufficient thermal energy to overcome band gaps or activation barriers, promoting excitations that enhance both conductivity and thermopower while initially outpacing thermal conductivity increases; this results in a pronounced monotonic rise in $ZT$ for temperatures exceeding 0.2 eV across all $g$ values, consistent with the activation of Dirac-like and flat-band states in pseudospin-1 systems where thermal broadening optimizes the energy window for efficient transport. However, beyond this threshold, $ZT$ diminishes with escalating $g$, as stronger coupling amplifies electron-phonon scattering rates–manifesting as increased polaron formation and quasiparticle damping–which suppresses $\sigma$ and $S$ more severely than it affects $\kappa$, thereby degrading overall thermoelectric performance.

**Fig. 14:**
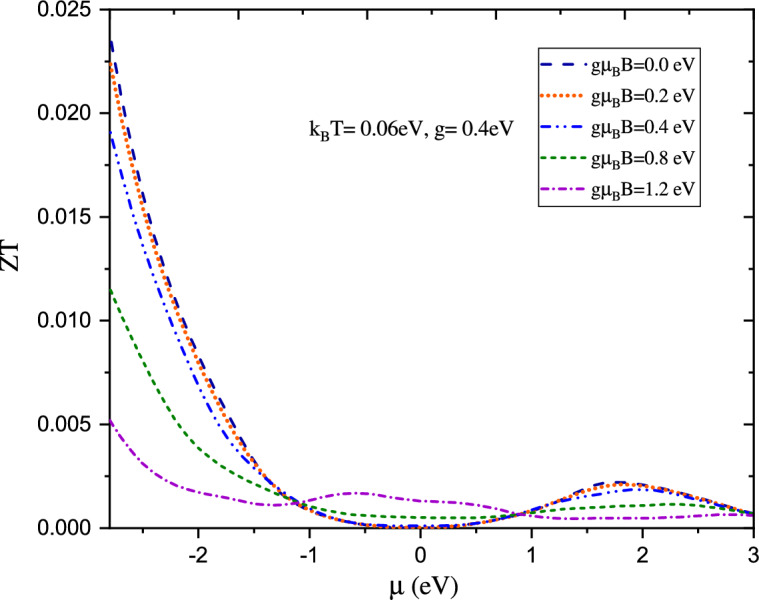
Thermoelectric figure of merit in a doped dice lattice plotted as a function of chemical potential $\mu$, evaluated for various magnetic field strengths such as $g\mu _B B = 0.0$ eV, 0.2 eV, 0.4 eV, 0.8 eV, and 1.2 eV, at a fixed temperature of $k_B T = 0.06$ eV, with the electron-phonon coupling strength set to $g = 0.4$ eV.

To elucidate the magnetic field’s influence on the figure of merit, Fig. 14 plots $ZT$ as a function of chemical potential under different strengths of $g \mu _B B$. In the doping regimes of $-3.0$ eV $< \mu < -1.0$ eV and $1.0$ eV $< \mu < 3.0$ eV, intensifying the magnetic field correlates with a decline in $ZT$. This decline is attributable to Zeeman splitting that symmetrizes electron-hole contributions, reduces the density of states asymmetry essential for high thermopower, and potentially enhances orbital quantization effects that localize carriers and elevate thermal losses. Conversely, within the near-half-filling interval of $-1.0$ eV $< \mu < 1.0$ eV, elevating the magnetic field augments $ZT$, offering a pathway for thermoelectric optimization. This enhancement likely stems from field-induced suppression of flat-band localization near the Dirac point, which sharpens the Fermi surface and boosts selective carrier transport without proportionally increasing $\kappa$. Additionally, for each magnetic field strength, $ZT$ exhibits a monotonic decrease with chemical potential in the deeply p-doped region of $-3.0$ eV $< \mu < -1.2$ eV. This decrease reflects carrier saturation where excessive doping intensifies scattering mechanisms, such as impurity or electron-electron interactions, that outweigh gains in conductivity.

Although $ZT$ decreases with increasing $g$ and $B$ in some regimes due to enhanced scattering, moderate magnetic fields enhance $ZT$ near half-filling ($-1.0$ eV $< \mu < 1.0$ eV) through Zeeman-induced asymmetry of the flat band, while Holstein phonons provide additional tunability of the effective mass. This synergistic control constitutes the central physical insight of the work.

We note that the present work computes only the electronic thermal conductivity $\kappa _e$. The lattice contribution $\kappa _\text {ph}$ is omitted, although stronger Holstein coupling is expected to suppress $\kappa _\text {ph}$ through enhanced phonon scattering. Future studies combining our electronic formalism with phonon Boltzmann transport are needed to obtain the total $\kappa$.

## Experimental implications and material design

Our results suggest that gate-tuned doping near half-filling combined with moderate perpendicular magnetic fields and controlled Holstein coupling can optimize $ZT$ in SrTiO$_3$/SrIrO$_3$ heterostructures. Microwave spectroscopy and strain engineering are promising routes to experimentally verify the predicted synergistic tunability. Similar to strategies proposed for LaCuSe$_2$ and related layered chalcogenides^58,83^, tensile strain engineering in dice-lattice heterostructures can further suppress $\kappa _\text {ph}$ and enhance overall $ZT$.

Regarding the orientation of the magnetic field, an in-plane field primarily induces spin-orbit-like effects without strong Zeeman splitting of the flat band. This leads to weaker modulation of the density of states near the Dirac point and consequently smaller enhancements in the Seebeck coefficient and $ZT$ compared to the perpendicular geometry studied here. The anisotropic response of thermoelectric parameters ($\sigma$, $S$, $ZT$) under in-plane versus perpendicular fields can therefore be exploited for directional control in future spintronic-thermoelectric devices. A full quantitative treatment requires future work.

## Conclusions

In summary, this work has systematically explored the impact of electron-phonon coupling and perpendicular magnetic fields on the electronic, transport, and thermoelectric properties of doped dice lattices using the Holstein model within a Green’s function formalism. The calculated density of states demonstrates a reduction at the Fermi level with increasing magnetic field strength, transitioning the system toward semiconductor behavior, while electron-phonon interactions further suppress the DOS at zero energy. Temperature-dependent analyses reveal that electrical conductivity decreases with rising temperature and electron-phonon coupling due to enhanced scattering, whereas thermal conductivity exhibits a peak followed by a decline, influenced similarly by these parameters. Thermoelectric metrics, including the Seebeck coefficient, power factor, Lorenz number, and figure of merit, show distinct dependencies on temperature, doping, magnetic field, and coupling strength, with notable improvements in figure of merit achievable in specific chemical potential regimes under magnetic influence. These insights highlight the potential for tailoring dice lattice properties through external fields and interactions, paving the way for applications in thermoelectric energy conversion and spintronic devices. Future studies could extend this framework to incorporate additional interactions, such as electron-electron correlations, to further refine predictive models for these intriguing two-dimensional materials.

## Data Availability

The datasets analyzed in this study are available from the corresponding author upon reasonable request.
